# Formaldehyde Adsorption–Desorption of Poplar Bark

**DOI:** 10.1007/s00128-019-02718-7

**Published:** 2019-09-24

**Authors:** Zoltán Pásztory, Katalin Halász, Zoltán Börcsök

**Affiliations:** 1 Innovation Center, University of Sopron, 4 Bajcsy Zs. str., Sopron, 9400 Hungary; 2 Natural Resources Research Center, University of Sopron, 4 Bajcsy Zs. str., Sopron, 9400 Hungary

**Keywords:** Formaldehyde, Adsorption, Desorption, Poplar bark

## Abstract

This study investigated how the *Populus* × *euramericana* cv. *Pannónia* bark behaves in an environment containing formaldehyde. Prism shaped samples were formed from the bark and the prisms were kept in formaldehyde contaminated atmosphere for 1, 2, 5, 10, 18 and 36 days. After the contamination, the amount of the formaldehyde adsorbed and later the desorbed was measured. The formaldehyde content of the uncontaminated poplar bark was 0.0036 mg/g. The amount of bound formaldehyde showed a saturation curve as a function of time. The formaldehyde adsorption reached an equilibrium value of 0.9 mg HCHO/g bark. The emission of formaldehyde from contaminated bark samples showed an exponential curve as a function of time and some residual formaldehyde content was detected after the formaldehyde was released.

As the indoor air-pollution problem of volatile organic compounds (VOC) like formaldehyde (HCHO) has arisen, over 500 VOC were detected (Chi et al. 2016; Plaisance et al. 2013). The adsorption of formaldehyde with activated carbon and various inorganic porous materials, carbon nitride nanotubes have been studied (Beheshtian et al. 2013; Boonamnuayvitaya et al. 2005; Jing et al. 2008). Extracts of bark are better examined in the literature (Chen 1982, 1991; Chen and Hatano 1990; Chen et al. 1991; Takano et al. 2008a), and there are only a few research studies focusing on the HCHO adsorption of bark itself (Funaki et al. 2004; Takano et al. 2008b). However, it was shown that using bark even as a bio-indicator can be advantageous, since it can adsorb gases from the air (Härtel 1982; Böhm et al. 1998; Saarela et al. 2005; Mandiwana et al. 2006). Numerous tree species such as oaks (*Quercus* sp.), elm (*Ulmus* sp.), willow (*Salix* sp.), poplar (*Populus* sp.), ash (*Fraxinus* sp.), maple (*Acer* sp.), lime (*Tilia* sp.), pine (*Pinus* sp.), yew (*Taxus baccata* L.), black locust (*Robinia pseudoacacia* L.) olive tree (*Olea europea* L.), cedar (*Cedrus atlantica* Endl.), cypress (*Cupressus sempervirens* L.), eucalyptus (*Eucalyptus* sp.) and others have been used to detect contaminants adsorbed from the air (Pásztory et al. 2016). Among them, poplar bark is also useful for detecting pollutants through adsorption (Berlizov et al. 2007). Due to its unique structure, the porous nature and the high extract content, bark can be a promising adsorbent material. Suberin and condensed tannins are important constituents of tree bark, but several other types of substrate materials can be observed in bark in different proportions such as waxes, terpenes, flavonoids, and alkaloids etc. (Bianchi 2017; Hathway 1958; Kurth 1947; Laks 1991; Narasimhachari and Rudloff 1961; Navarrete et al. 2011). Tannin in bark, having different types of flavan-3-ol units (Porter 1992; Schofield et al. 2001), can react with formaldehyde vapor (Pizzi 1980, 2003).

The (+)-catechin is usually used as model compound to study the reaction of condensed tannins with formaldehyde (Hemingway and McGraw 1978; Hillis and Urbach 1959; Kiatgrajai 1978; Mater 1955; Saito et al. 2001; Takagaki et al. 2000). Takano et al. (2008a) studied the reaction of wood bark (+)-catechin and formaldehyde and found it can occur without a solvent or catalyst, similarly as in an aqueous solution. During the reaction, methylolation and condensation can form, and after that polymerization can also take place.

Takano et al. (2008b) examined the formaldehyde adsorption of milled and ground karamatsu (*Larix leptolepis*) bark with different particle sizes; and solvent extracted bark fractions. Two different particle size ranges (60–100; <100 mesh) were examined but the influence of the size of the particle of the bark sample on formaldehyde adsorption was relatively small. They found that the milled and ground bark had a higher HCHO adsorption than the microcrystalline cellulose used as a reference material. They reported that the acetone-soluble fraction had a high adsorption, but minor desorption of formaldehyde, which was attributed to its high tannin content. It was concluded that an aldol-condensation-type reaction was responsible for formaldehyde chemical adsorption.

Hybrid poplars are 14.5% of the trees harvested in Hungary (NÉBIH 2016), and the importance of poplar plantations is expected to increase, thus increasing the amount of the harvested wood and the available bark. The overall objective of this study was to determine the rate of formaldehyde adsorption and emission characteristics of the poplar bark and to investigate the amount of bound formaldehyde during and after contamination. Determination the remaining bonded formaldehyde was also one of the goals of this examination.

## Materials and Methods

Poplar (*Populus* × *euramericana* cv. *Pannónia*) prisms were cut from bark slabs containing only the outer bark. Bark was collected from the local sawmill in Sopron, Hungary. Bark was peeled from wet timber to be cut and was dried to around 15% moisture content before the formation of prisms. The inner bark was separated from the bark slabs when the prisms were formed; the sizes of the outer bark prisms were 3 × 2.5 × 2 cm^3^. Prisms were stored for one month at 20°C and 65% relative humidity. The experiment started when the weight of the prisms was constant for 7 days.

A formaldehyde solution (37%), toluene, acetyl acetone and ammonium acetate which was used for the determination of adsorbed formaldehyde was obtained from Molar Chemicals Hungary Ltd.

For the adsorption test, the formaldehyde was diluted with distilled water to 2:10. 20 mL solution was added to a crystallizing dish, which was placed in a glass desiccator (11 L). Two hundred grams of bark prisms were placed on a stainless-steel wire above the dish before the desiccator was closed and sealed. The desiccator was kept in a climate chamber at 65 RH% and 23°C. After the tests (1, 2, 5, 10, 18 and 36 days) the samples were halved; one half was used to determine the formaldehyde content of the bark prisms and the other half was placed in a desiccator to measure the formaldehyde desorbed from the prisms.

The amount of adsorbed formaldehyde was measured by the perforator method according to MSZ EN ISO 12460-5:2016 (MSZ EN ISO 12460-5:2016 2016). In brief, a specified amount of sample was extracted with hot toluene in a glass perforator; the solvent was then bubbled through distilled water which adsorbs the extracted formaldehyde. The formaldehyde content is determined by a Hantzsch reaction, where acetyl acetone and ammonium acetate react with formaldehyde and form diacetyl dihydrolutidine. Its absorbance is read at 412 nm and the concentration is calculated based on the calibration curve. The calibration curve was determined with a formaldehyde standard solution (15 µg/mL, concertation calculated by titration with standardized sodium thiosulphate), at concentrations of 0, 0.15, 0.3, 0.75, 1.5, 3.0, 7.5 and 15 µg/L. The absorbance was plotted against the concentration values. R^2^ value of the fitted calibration curve was 0.9989. The perforator value (the formaldehyde content) was expressed in mg HCHO/g bark.

The desorption test was based on the desiccator method described by MSZ EN ISO 12460-4:2016 (MSZ EN ISO 12460-4:2016 2016) with a few modifications regarding the test duration, sample appearance and calculation. The test was performed in the same way as the adsorption test except that the crystallizing dish contained 300 mL of distilled water to adsorb the formaldehyde released from the prisms. During the test, a two times 5 mL sample from the distilled water was collected and used for the determination of the formaldehyde emission by a Hantzsch reaction. A calibration curve was used to calculate the concentration. The released formaldehyde was expressed in mg HCHO/g bark. All the tests were performed in triplicate.

## Results and Discussion

**Fig. 1:**
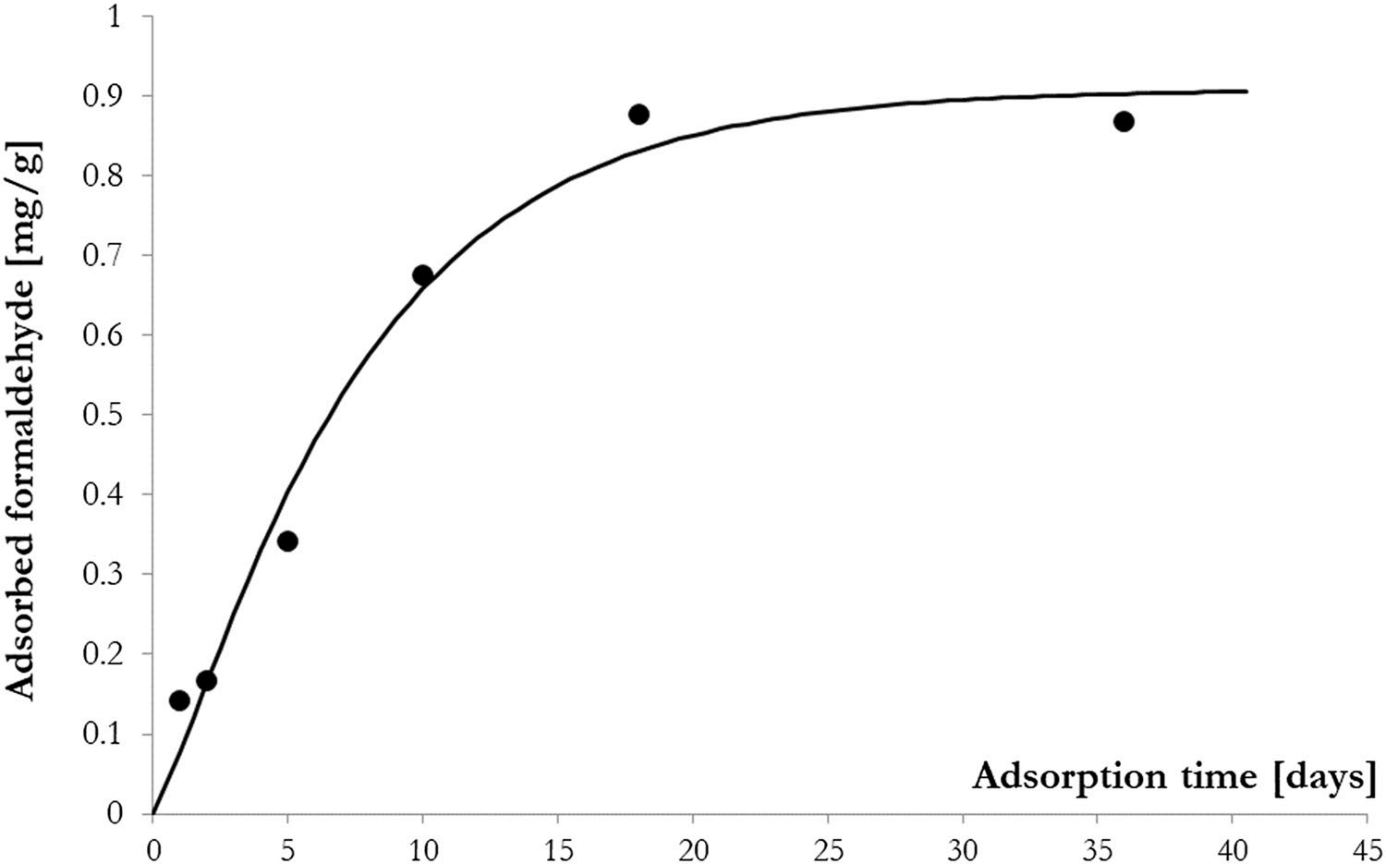
Formaldehyde adsorption by poplar bark samples in a function of time (the data points are mean of three measured values, standard deviation < 0.02)

In a polluted environment, bark samples adsorbed formaldehyde from the atmosphere. The longer the bark samples were in the polluted environment, the more formaldehyde was bound. The bark samples continuously adsorbed formaldehyde from the atmosphere in the first few days and it slowed after ten days (Fig. 1). The formaldehyde sorption of bark can also be physical and chemical. The gas molecules can be adhered to the surface without chemical bonding due to the highly porous surface of the bark. The outer bark contains small pores called lenticels which enables gas exchange from the inner layers (Groh et al. 2002). The cracked, folded structure of the bark and the lenticels provide a high specific surface area and thus abundant adsorption sites (Chrabąszcz and Mróz 2017). The higher porosity of the outer bark is associated with greater hygroscopicity (Ilek et al. 2016), thus allowing it to physically bind larger amounts of material. Due to the high extract content of bark (like tannins), chemical sorption can also take place in the HCHO binding (Takano et al. 2008a, b).

The whole process is characterized by a saturation curve since the number of "sites" found in the cortex for the formaldehyde is finite, and the bound formaldehyde molecules loaded them over time (Fig. 1 and Eq. 1). Based on the shape of the fitted curve, the bark follows the adsorption profile of microporous materials (Lawrence and Jiang 2017). We found that the Pannónia poplar bark can adsorb 0.9 mg/g of formaldehyde.

1 $$y=0.908003\cdot {\left(1-{e}^{-0.149019\cdot x}\right)}^{1.25925}\left[{R}^{2}=0.99\right]$$

Formaldehyde emissions of uncontaminated bark sample was also investigated, and 0.0036 mg/g poplar bark was measured, which value is significantly lower than that of the adsorbed amount of formaldehyde.

**Fig. 2:**
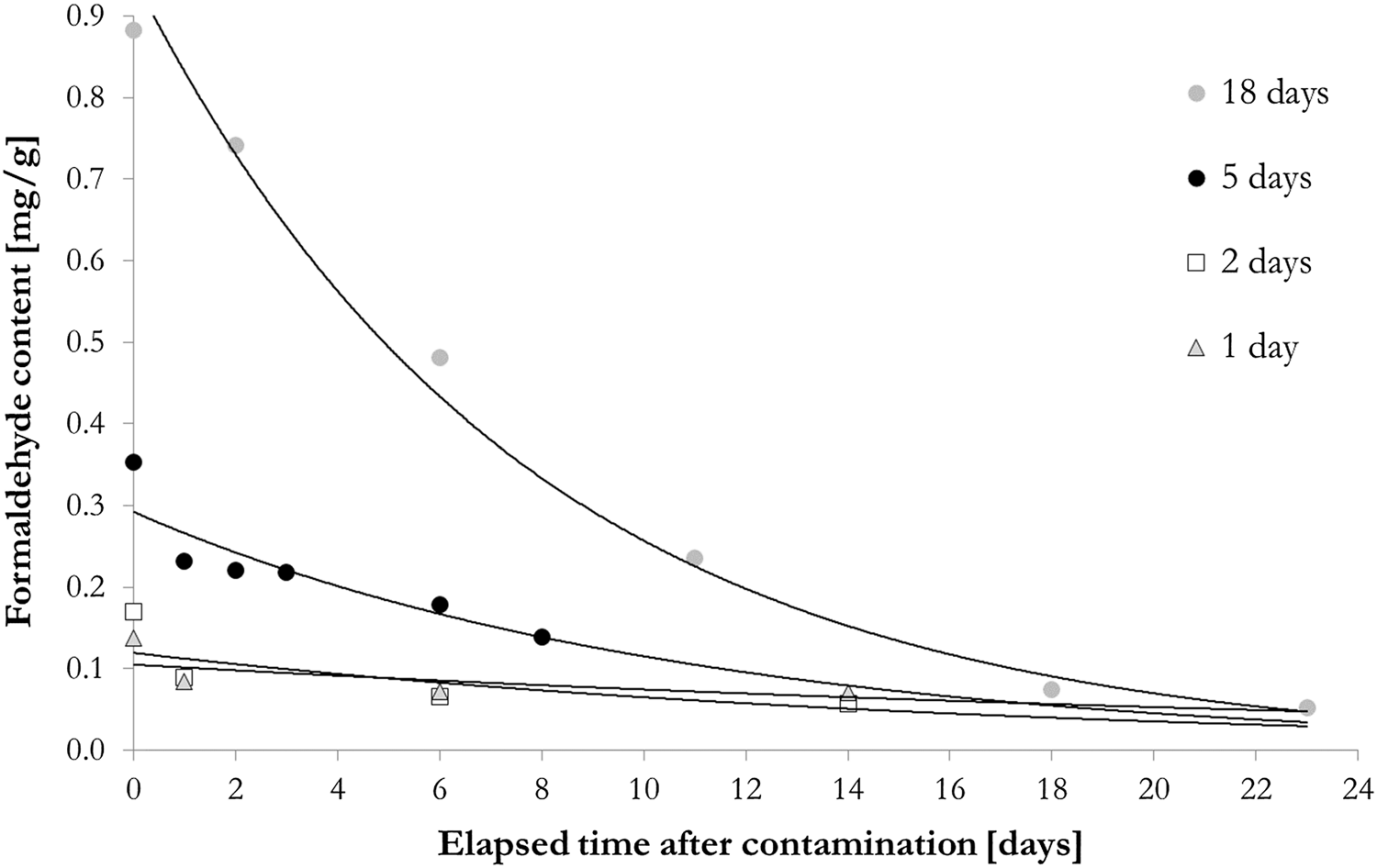
Decrease of formaldehyde content of contaminated poplar bark samples over time (the data points are mean of three measured values, standard deviation < 0.03)

The release of formaldehyde from bark samples kept for 1, 2, 5 and 18 days in a polluted environment was investigated after the termination of the contamination stopped. Polluted samples started to release the formaldehyde immediately after the contamination stopped. This indicates that the adsorption of the formaldehyde is dominantly physisorption with relatively weak interactions between the gas molecules and the bark surface. As time progressed, less formaldehyde remained in the samples but it could be detected after long periods of time (Fig. 2 and Eqs. 2–5 for 1, 2, 5 and 18 days respectively).

2 $$y=0.1094\cdot {e}^{-0.033\cdot x}\left[{R}^{2}=0.49\right]$$

3 $$y=0.1184\cdot {e}^{-0.06\cdot x}\left[{R}^{2}=0.65\right]$$

4 $$y=0.2922\cdot {e}^{-0.093\cdot x}\left[{R}^{2}=0.86\right]$$

5 $$y=0.9426\cdot {e}^{-0.13\cdot x}\left[{R}^{2}=0.99\right]$$

The remaining formaldehyde was 15 to 20 times more than could be detected from the uncontaminated samples, which were between 0.048 and 0.075 mg/g. This result shows that although physisorption is the main mechanism, chemisorption could also play a role in the formaldehyde immobilization. Chemical bonds might form between the HCHO molecules and the surface functional groups of the poplar bark components.

In summary, the current study found that in a formaldehyde polluted environment, the Pannónia poplar bark prisms can adsorb detectable formaldehyde amounts from contaminated air. The amount of adsorbed formaldehyde depends on the length of exposure and shows a saturation curve as a function of time. The results show that most of the adsorbed formaldehyde is bound physically and reversibly and after the contamination, the poplar bark emits the adsorbed formaldehyde. The emission of the formaldehyde from the bark prisms shows an exponential curve as a function of time. In the contaminated poplar bark prisms, a formaldehyde residue can be detected even after formaldehyde was released, which is greater than the amount of formaldehyde that can be detected from uncontaminated bark, indicating a chemical adsorption as well. There is a continuous need to discover new natural based materials that are able to adsorb harmful compounds from polluted air. Bark is a natural, renewable and available in large quantities form wood processing at low cost. Based on our results poplar bark can be applied as a regenerable formaldehyde adsorbent even in prism form. In the last case, the bark contaminated with formaldehyde can be disposed of by burning in a carbon neutral way for heat production.
